# Milestones in the Evolution of Hepatic Surgery

**DOI:** 10.5041/RMMJ.10021

**Published:** 2011-01-31

**Authors:** Henri Bismuth, Rony Eshkenazy, Arie Arish

**Affiliations:** 1 Hepatobiliary Institute, Paul Brousse Hospital, Paris, France, and; 2 Hepato-Biliary Surgery Service, Department of General Surgery, Rambam Health Care Campus, Haifa, Israel

**Keywords:** Surgery, liver, transplantation, transection

## Abstract

This paper describes the rapid evolution of modern liver surgery, starting in the middle of the twentieth century. Claude Couinaud studied and described the segmental anatomy of the liver, Thomas Starzl performed the first liver transplantations, and Henri Bismuth introduced the concept of anatomical resections. Hepatic surgery has developed significantly since those early days. To date, innovative techniques are applied, using cutting-edge technologies: Intraoperative ultrasound, techniques of vascular exclusion of the liver, new devices for performing homeostasis and dissection, laparoscopy for resections, and new drugs that allow the resection of previously unresectable tumors. The next stage in liver surgery will probably be the implementation of a multidisciplinary holistic approach to the liver-diseased patient that will ensure the best and most efficient treatments in the future.

The adult liver, the largest organ in the body, accounts for 2%–3% of overall body weight. Richly vascularized, the liver has an inflow carried through the portal vein and the hepatic artery and an outflow draining through the main and accessory hepatic veins. The main portal vein drains the splanchnic territory and carries 70%–80% of the overall hepatic inflow. It divides into two branches, the right and left portal veins, which are then subdivided into sectorial and segmental branches. Blood pressure in the main portal vein is usually low, with a portocaval gradient (i.e. portal vein pressure minus central venous pressure) of less than 5 mmHg. In chronic liver disease, especially cirrhosis, the portocaval gradient may increase to the point of portal hypertension.

In the seventeenth and eighteenth centuries, liver resections were carried out on trauma victims with a range of injuries occurring as a result of military combat. The first liver resections in a non-emergency setting resulted in uncontrolled bleeding and death.

## HISTORY OF MODERN LIVER SURGERY

The German surgeon Carl Johann August Langenbuch performed the first successful hepatic resection in 1888 (he was the first to perform cholecystectomy in 1882).^1^ He resected a part of the left lobe of the liver after ligating the vascular pedicles. The pathologic examination of the specimen revealed normal liver.

The first steps leading to modern liver surgery began in the late 1950s. Scattered publications from the United States describing a limited series of liver resections that had met with some success were published. The technique of liver resection at that time was ill defined.^2^,^3^

Concomitantly, in 1952 in France Lortat Jacob published a manuscript on his experiences performing anatomical liver resections: right hepatectomy after vascular control of the right liver.^4^ In 1956, Claude Couinaud,^5^,^6^ after studying casts of the liver, published his book, *The liver – anatomicalstudies and surgical studies*. He showed that, according to the distribution of the portal blood, the liver contains four parts that are subdivided by the hepatic veins into eight segments. He coined the segment numbers as follows: one for the central segment and two through eight for the seven other segments in a clock-wise fashion. This segmental division of the liver is the basis of modern functional and surgical liver anatomy.

Despite the anatomical discoveries of the 1950s, their application in surgical practice was limited. No clinical methods existed that could detect the existence of small liver tumors that might have required segmental resections. Physicians made their diagnoses based on physical symptoms (pain or a palpable tumor), at which point it was usually already too late for surgery. Only large liver resections could be performed.

The introduction of ultrasound in the early 1980s^7^,^8^ into common clinical practice allowed the clinician to diagnose asymptomatic small liver tumors of 2 and 3 cm and paved the way to rapid development of liver surgery. In fact, modern liver surgery began when functional liver anatomy discovered 30 years earlier was applied, enabling segmental liver surgery.

The paper with the somewhat provocative title of “Surgical anatomy and anatomical surgery of the liver”^9^ published in 1982 was a turning-point in the practice of liver surgery. Navigating surgery on the basis of anatomy eliminated the use of “atypical” or “non-anatomical” resections of the liver which had resulted in bleeding, increased morbidity, and mortality sometimes as a result of liver necrosis. Liver resections based on anatomy gained popularity and evolved into bloodless surgery that removed independent segments or groups of two or more segments.

**Figure 1:**
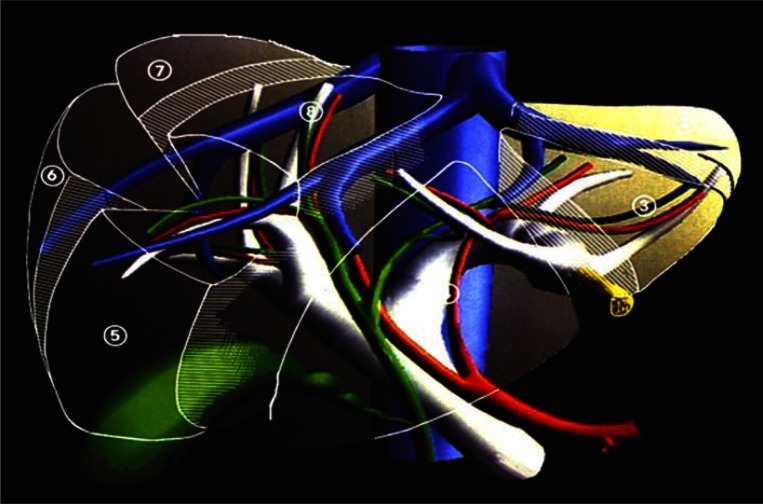
The transparent liver showing the main arteries (in red), portal vein (in white), hepatic veins (in blue), and main parts of the biliary system (in green). Liver segments are numbered, and the round ligament is designated in yellow.

In 1984, intraoperative ultrasound (IOUS) was introduced into practice.^10^–^14^ The technique allowed the surgeon to understand liver vasculature and biliary duct anatomy and rendered the liver transparent (Figure 1). In fact, the introduction of IOUS into liver surgery had the same impact as the inclusion of intraoperative cholangiography (IOC) fifty years earlier in biliary surgery.^15^ By finding the liver’s lines of division with IOUS and *looking* for a tumor, the surgeon could establish the relations among the portal elements, the hepatic veins, the hepatic parenchyma, and the tumor, and then decide what kind of anatomical resection needed to be carried out – “*Hepatectomie à la carte*” for each individual patient.

Control of bleeding during liver resection is a major challenge for the surgeon. It is particularly difficult in cirrhotic liver due to the fibrotic nature of the liver tissue. The indication, as well as the type of clamping, depends mainly on the size and location of the lesions to be resected, the quality of the liver parenchyma, the surgeon’s preferences, and unexpected operative events. In 1958 Lin introduced the finger fracture technique, which involves crushing of liver parenchyma by the surgeon’s fingers under inflow occlusion so as to isolate vessels and bile ducts for ligation.^16^ This technique was subsequently improved through the use of surgical instruments such as a small Kelly clamp for blunt dissection (clamp crushing or “Kellyclasia”).^9^,^17^,^18^

In many centers, ultrasonic dissection using the Cavitron Ultrasonic Surgical Aspirator (CUSA; Tyco Healthcare, Mansfield, MA, USA) has become the standard technique for liver parenchyma dissection. With this technology, the liver tissue is fragmented with ultrasonic energy and aspirated, thus exposing small vascular and ductal structures that can be ligated or clipped with titanium hemoclips.^19^ The water jet dissector employs a pressurized jet of water instead of ultrasonic energy, in order to fragment the liver parenchyma tissue and expose the vascular and ductal structures.^20^ However, this technique has not become as popular as ultrasonic dissection.

**Figure 2:**
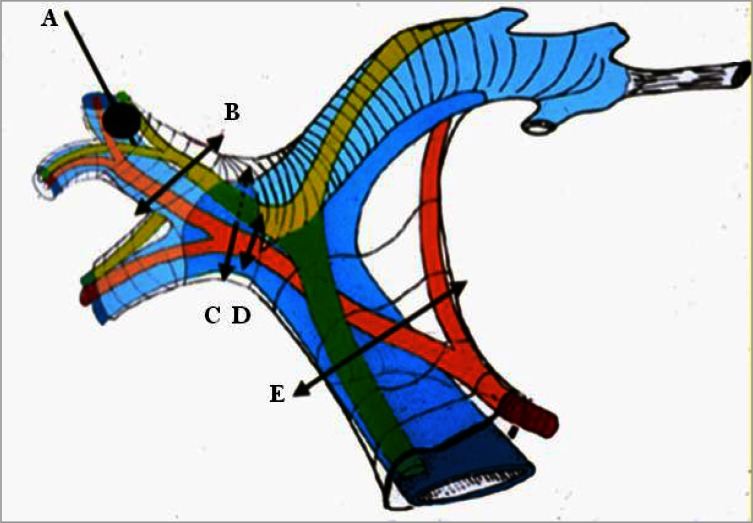
Glissonian pedicle elements: portal vein (in blue), hepatic artery (in red), and the bile ducts (in green). Hepatic inflow occlusion: A) Selective occlusion of segmental portal vein by a balloon introduced under ultrasonographic guidance. The arrows show the different sites of Glissonian clamping. B) Suprahilar clamping of one sector of the right liver; C and D) hilar selective clamping to the right liver; E) total pedicular clamping (Pringle maneuver).

To reduce blood loss during liver resection, intermittent inflow occlusion by clamping of the portal triad (Pringle maneuver) is frequently used.^21^,^22^ However, there is a limit to how long the Pringle maneuver can be applied. Prolonged inflow occlusion (over 120 minutes) may have deleterious effects on liver functions.^23^ Hepatic inflow occlusion can be directed to one side or to a segment by clamping the Glissonian pedicle at the hilum or inside the liver parenchyma (Figure 2).^9^,^24^–^29^

**Figure 3:**
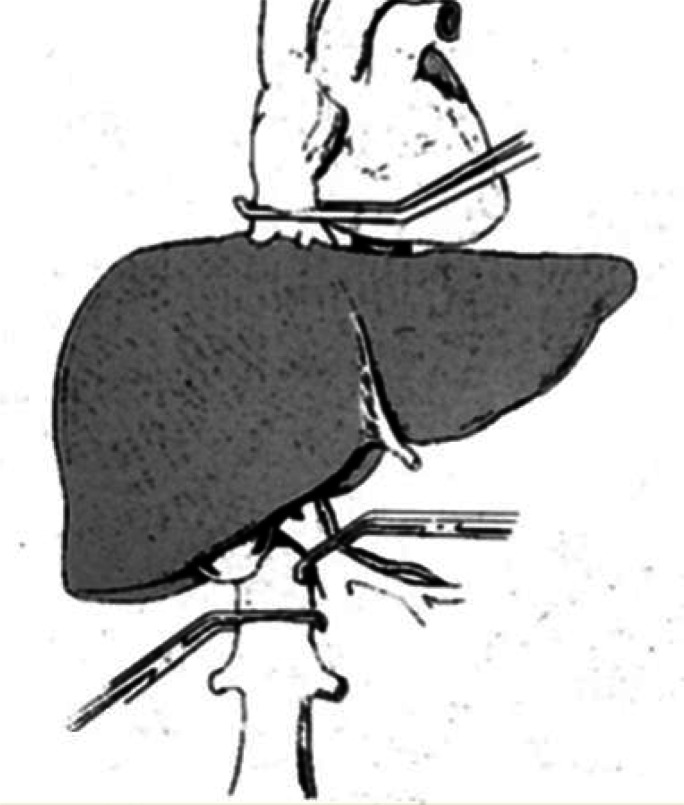
Total vascular exclusion of the liver by clamping the infrahepatic and suprahepatic inferior vena cava and the hepatoduodenal ligament.

**Figure 4:**
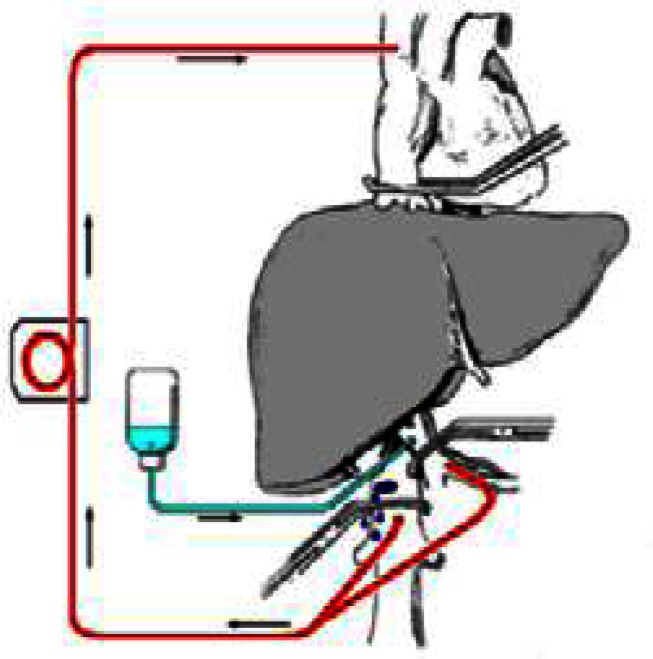
Total vascular exclusion of the liver with hypothermia as described by Fortner et al.^40^ The liver is excluded (as in Figure 3). Veno-venous bypass of the liver is performed (red lines), and hypothermic solution is infused into the portal vein (in blue).

**Figure 5:**
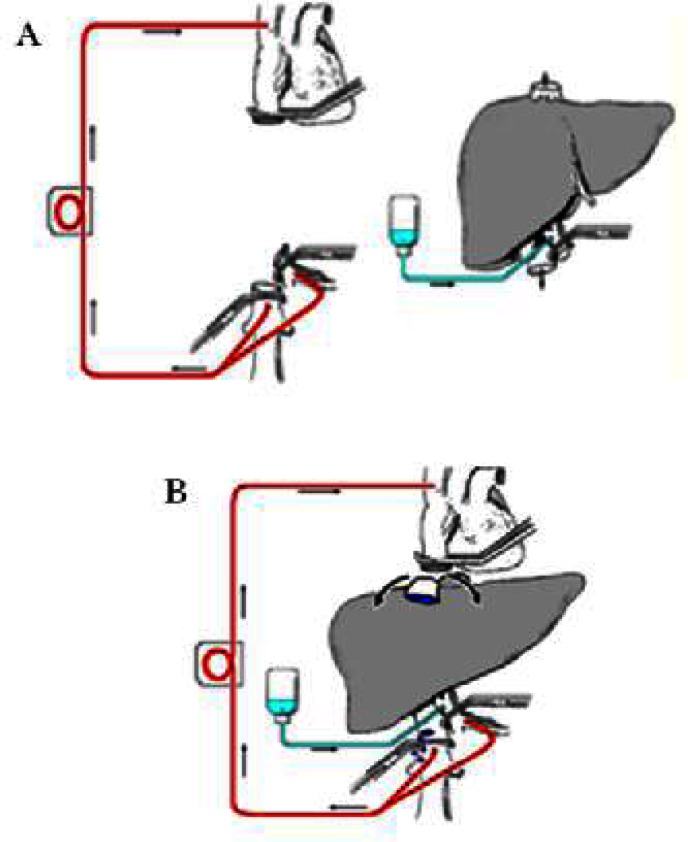
Total vascular exclusion for complex liver resections. A) The *ex-situex-vivo* technique described by Pichlmayr et al.^46^ B) The *ex-situin-vivo* technique described by Hannoun et al.^43^

The majority of liver resections can be done with no clamping at all.^30^,^31^ In some patients total hepatic vascular isolation is needed. This isolation can be partial (meaning occlusion of the inflow and only one hepatic vein^32^,^33^) or total (meaning occlusion of the inflow). Outflow occlusion is obtained by occluding the vena cava above and below the hepatic veins^34^–^37^ (Figure 3). In cases of a small tumor adherent to a hepatic vein, isolation of the corresponding liver by clamping the inflow and the hepatic vein can facilitate and render surgery safe if the lesion is resected with the adherent vein. When a large tumor is found to have entered the vena cava, this technique enables bloodless resection of the involved vena cava and safe reconstruction of its continuity.^38^,^39^ Total vascular exclusion (TVE) of the liver can be applied safely for as long as 60 minutes. This can be extended to 8 hours by using hypothermia (Figure 4), as is done in liver transplantation. The liver resection can be done *in situin vivo* as first described by Fortner et al.,^40^–^42^*ex situ in vivo* as described by Hannoun et al.,^42^–^45^ or *ex situ ex vivo* as described by Pichlmayr et al.^46^ (Figure 5, next page). This innovative approach to liver resection has a high rate of complication and even mortality.

Another concept in liver surgery that guides surgeons toward safe surgery is that “the liver is parenchyma tissue with blood vessels inside”. The rationale behind this concept is to dissect this parenchyma tissue while ensuring hemostasis. The concept was popularized following the development of new devices that enabled such dissection, e.g. the harmonic scalpel,^47^–^49^ LigaSure,^50^–^52^ tissue link,^53^ radiofrequency,^54^,^55^ and the “Habib sealer”.^56^,^57^ These devices allow the parenchyma to be cut without having to clamp the pedicle. Nevertheless, the clamp crushing technique is still widely used.^58^–^61^

The main indication for liver resection today is liver metastasis resulting from advanced cancers of the colon and rectum. Apart from liver surgery for trauma or hepatocellular carcinoma in cirrhotic patients where the mortality is high,^62^ the overall operative mortality in liver resections is between 0% and 2%.^63^,^64^ Trained liver surgery teams can achieve less than 1% mortality. This is a great advance in comparison to the mortality in liver surgery in early reports, which reached a mortality rate as high as 20%.^65^

## LIVER SURGERY – A NEW SPECIALTY

In medicine and surgery, the use of special technologies and knowledge has facilitated the development of subspecialties. Cardiac surgery, for example, emerged as a specialty of surgery because of the use of special machines and instruments. This was not the case for liver surgery.

When it began, liver surgery used nothing but the instruments of general surgery. Today, with the exploitation of special technology and instrumentation such as operative ultrasound, ultrasonic dissectors, argon coagulators, cryotherapy, radiofrequency, and extracorporeal circulation, liver surgery has become a subspecialty of general surgery, or more precisely, a *hyper-specialty.*

One step on the way to liver surgery becoming a specialty was the advent of liver transplantation. When liver surgeons began performing transplantation, liver resection was a step in the procedure. Surgery on the graft itself or on the recipient, which may have meant heterotopic liver transplantation and reducing the size of the graft,^66^,^67^ operating on the living donor,^68^ performing a domino transplantation,^69^,^70^ or a split liver procedure,^71^–^74^ were all done by liver surgeons doing liver transplantation.

Liver surgery is now a specialty, as one of the three branches of gastro-intestinal (GI) surgery. These are upper GI, colorectal, and hepato-biliopancreatic surgery. Moreover, it is not a narrow field of surgery. Today’s liver surgeons need to master a variety of techniques and tactics: open liver surgery, laparoscopic surgery, endoscopic surgery, and percutaneous surgeries (minimal access surgery).

The two branches, liver surgery and liver transplantation, overlap:

- Techniques of liver transplantation in liver surgery: External bypass in extensive resections, cooling (topical, hypothermia, *ex-situ* resections).
- Techniques of liver surgery in liver transplantation: Reduced size graft, split liver, living donor.

Today’s liver surgeon needs to be familiar with anatomy, echography, vascular surgery, microsurgery, immunology, hepatology, and oncology.

## THE PRESENT

Improving the technique of liver resection and increasing the number of patients eligible for liver resection are the main objectives because the only way to cure a patient, in cases of liver tumors, is to offer him or her the chance of a liver resection.

There are two main ways to render a patient eligible for liver resection: by changing the tumor size through chemotherapy or changing the remaining liver size by performing portal vein embolization.^75^–^80^

In cases of multiple, bilateral, unresectable colorectal liver metastases, the strategy must be multimodal, starting with neoadjuvant chemotherapy. In patients with a normal liver, portal vein embolization (PVE) is indicated when the future liver remnant volume is predicted to be <30%.^81^–^95^ PVE may also be useful in patients who have evidence of chemotherapy-related liver injury. If there is a positive response to chemotherapy, then surgery should follow. Limited resection of the colorectal liver metastases in one lobe can be performed as the first stage combined with radio frequency ablation of other lesions and PVE of the contralateral portal vein. Alternatively, ligation and alcoholization of the portal vein can be performed intraoperatively. PVE, ligation, and/or alcoholization produce, on the one hand, essential atrophy of the implicated lobe and, on the other hand, significant hypertrophy of the contralateral lobe. This strategy enhances the volume of the future liver remnant. Studies have shown an approximately 10%–25% increase in the size of the liver remnant after PVE.^81^–^95^ The hypertrophy is complete by the third week. In a second stage, a major resection of the contralateral liver can be carried out safely.^96^–^98^ Usually, patients undergoing these procedures receive postoperative chemotherapy. With this approach, currently there are no limits to the resection other than those imposed by the volume and function of the liver remnant.

Is laparoscopic liver surgery an abdominal approach or a technical improvement? It is too early to compare laparoscopic and open surgery since there are much fewer laparoscopic liver resections reported^99^,^100^ than open liver resections. Many thousands of open liver resections are performed every year. Laparoscopic liver surgery is feasible and safe for certain indications, e.g. left lateral segmentectomy for benign or malignant disease.^101^–^106^ Harvesting of the left lateral segment for living related transplantation is done by laparoscopy by some expert surgeons (experts in liver surgery, liver transplantation, and laparoscopy)with excellent results.^107^

There are some reports on robotic liver resections, though these are still preliminary descriptions.^108^

## THE FUTURE

The future of liver surgery and new developments in the field are moving in a multidisciplinary direction. Today, oncologists, hepatologists, surgeons, endoscopists, radiologists, pathologists, and researchers are all members of the team treating patients. Their joint efforts are intended to give patients the best and most efficient treatment. In the coming years, the positive results of this co-operation should lead to new and successful treatment procedures.

## Abbreviations:

IOC: intraoperative cholangiography;
IOUS: intraoperative ultrasound;
PVE: portal vein embolization.
